# Healthcare workers’ behaviors and personal determinants associated with providing adequate sexual and reproductive healthcare services in sub-Saharan Africa: a systematic review

**DOI:** 10.1186/s12884-017-1268-x

**Published:** 2017-03-13

**Authors:** Kim Jonas, Rik Crutzen, Bart van den Borne, Priscilla Reddy

**Affiliations:** 1 Department of Health Promotion, School of Public Health and Primary Care (CAPHRI), Faculty of Heath, Medicine and Life Sciences, Maastricht University, P.O. Box 616, 6200 MD Maastricht, The Netherlands; 2 Faculty of Community and Health Science, University of the Western Cape, Cape Town, South Africa; 3 Human Sciences Research Council (HSRC), Population Health, Health Systems and Innovation Unit, Cape Town, South Africa

**Keywords:** Healthcare worker behaviour, Personal determinants, Sexual and reproductive healthcare, Adolescent health, Maternal health, Child health, Healthcare services, Systematic review

## Abstract

**Background:**

Healthcare workers may affect the utilization of sexual and reproductive healthcare (SRH) services, and quality of care thereof, for example by their behaviours or attitudes they hold. This can become a hindrance to accessing and utilizing SRH services, particularly by young people, and thus a better understanding of these behaviours and associated factors is needed to improve access to and utilization of SRH services.

**Methods:**

A systematic review of literature was conducted to identify studies focusing on healthcare workers’ behaviors and personal determinants associated with providing adequate SRH services in sub-Saharan Africa (January 1990 - October 2015). Five databases were searched until 30th October 2015, using a search strategy that was adapted based on the technical requirements of each specific database. Articles were independently screened for eligibility by two researchers. Of the 125-screened full-text articles, 35 studies met all the inclusion criteria.

**Results:**

Negative behaviours and attitudes of healthcare workers, as well as other personal determinants, such as poor knowledge and skills of SRH services, and related factors, like availability of essential drugs and equipment are associated with provision of inadequate SRH services. Some healthcare workers still have negative attitudes towards young people using contraceptives and are more likely to limit access to and utilization of SRH by adolescents especially. Knowledge of and implementation of specific SRH components are below optimum levels according to the WHO recommended guidelines.

**Conclusions:**

Healthcare workers’ negative behaviours and attitudes are unlikely to encourage women in general to access and utilize SRH services, but more specifically young women. Knowledge of SRH services, including basic emergency obstetric care (EmOC) is insufficient among healthcare workers in SSA.

**Trial registration:**

A protocol for this systematic review was registered with PROSPERO and the registration number is: CRD42015017509.

**Electronic supplementary material:**

The online version of this article (doi:10.1186/s12884-017-1268-x) contains supplementary material, which is available to authorized users.

## Background

Sexual and reproductive healthcare (SRH) and rights of women, including adolescents was placed on the international agenda in 1994, setting the tone for their priority in public health [1, 2]. Following the millennium development goals (MDG), SRH services are now further prioritized in the sustainable development goals (SDG), more specifically SDG target 3.7, which aim to achieve universal access to sexual and reproductive healthcare services by 2030 [3]. However, SRH needs for many women, particularly adolescents are still not met despite these global agreements. SRH services remain grossly inadequate, especially in low- and middle-income countries (LMICs) [2]. This inadequacy is congruent with high rates of maternal mortality and morbidity, infant and child mortality, sexually transmitted infections (STIs), including HIV, unwanted pregnancies particularly among adolescents, and unsafe and illegal termination of pregnancy (TOP). Consequently, the burden of SRH related diseases and mortality is high in LMICs, especially in sub-Saharan Africa (SSA) [4, 5].

SSA still remains the region grossly affected by maternal deaths at 201 000 in 2015 with more than 60% of all maternal deaths worldwide, being ascribed to the region [4–6].

SSA is amongst the regions affected by a high number of maternal mortality at 201 000 in 2015 and was reported to have contributed to 62% of all maternal deaths worldwide in 2013 [4–6]. Maternal mortality rates in SSA are the worst in the world, with 640 deaths per 100,000 live births [5, 6]. Of course, a variety of factors play a role in the different countries of SSA, such as the living conditions, poverty, healthcare systems of each country, burden of other diseases like HIV and AIDS, and cultural and religious practices. Caution therefore needs to be exercised when interpreting the healthcare services and outcomes from the different countries and when planning interventions for the improvement of SRH service provision. High maternal and child mortality rates in SSA are thought to be primarily due to a lack of resources; and failures of the healthcare system, poor pre- and post- natal care attendance, unsafe TOPs, especially among adolescents. For example, only 25% of postpartum mothers and newborns in SSA receive postnatal care within 48 h of birth [7]. Women in SSA face a 1 in 39 risk of dying in childbirth; and over 800 women die every day due to complications in childbirth and pregnancy [5]. Adolescents are at higher risk for maternal mortality, including infant and child mortality compared to older women due to many reasons, such as incomplete physiological development and, their SRH needs and rights still remaining non-prioritized in many SSA countries [5, 8].

Teenage pregnancy in SSA is the highest in the world, with more than half of all births occurring in this region [5, 8, 9]. The second worldwide leading cause of death among the 15-19 year old adolescent girls is complications during pregnancy and childbirth, with adolescent girls below the age of 16 years at an even higher risk for these complications, and consequently death and severe morbidity compared to women above the age of 20 years [5]. Given these alarming SRH outcomes in SSA, a quest to understand how healthcare workers provide SRH services and what factors are associated with adequate provision of the services was borne.

SRH services are often provided in the public health settings, although not always readily available in many resource-constrained settings like those of SSA, due to a number of environmental and socio-demographic factors. Such factors include lack of essential drugs and equipment, distance and long travel times to the facilities, shortage of healthcare workers and long waiting times at the facilities. Nurses and midwives are the healthcare professionals at the forefront in many public health facilities and are the most common category of healthcare workers women and adolescents consult for their SRH needs. When healthcare workers are available, they are often under-utilized especially by adolescents for various reasons, such as healthcare workers’ negative behaviors and attitudes [10–13].

Negative behaviours and attitudes of healthcare workers potentially affect access to and utilization of SRH services by women and adolescents especially, and the quality of care thereof. For example, studies in SSA indicate that healthcare workers’ negative behaviours discourage women from seeking antenatal care, and young people from attending clinics or follow-up visits [11–15]. Basic emergency obstetric care (EmOC), a component of SRH services, may also be significantly under-utilized by young people, especially adolescents who become pregnant during their adolescent years [13–15]. Hence, an in-depth understanding of healthcare workers’ behaviours and factors associated with their behaviours is needed in order to improve access to and utilization of SRH services at large, and by young people [4, 11, 16, 17].

Lack of respect for women’s opinion and preferences for birthing options for example, including adolescents’ privacy and confidentiality, and the ill treatment by healthcare workers were some of the reported negative behaviours that discouraged women from giving birth at the healthcare facility, and sexually active adolescents from seeking SRH services [11, 18, 19]. Adolescents also face difficulties obtaining contraceptives at public health facilities due to healthcare workers’ negative attitudes associated with the general social stigma towards adolescent who seek contraceptive services in SSA [11, 12, 19, 20].

The availability, accessibility, and utilization of SRH services may significantly alleviate the high rates of maternal mortality and morbidity, teenage pregnancy, STIs and HIV, unsafe TOPs, and infant and child mortality and morbidity in SSA. However, in order to accomplish these promising health outcomes in SRH services, healthcare workers need to adequately provide these services to women and adolescents in need, without any prejudice and limitations. Adequate provision of SRH services to young people particularly, encompasses amongst others, offering a youth-friendly environment, possessing a positive attitude towards the young people who use the services, being knowledgeable about their SRH issues and needs, and a willingness to serve them.

Therefore, the objective of this systematic review was to determine healthcare workers’ behaviours and related personal determinants associated with providing adequate and quality SRH services to women and adolescents in sub-Saharan Africa. To achieve this objective, this review sought to answer the following research questions: 1) which healthcare workers’ behaviours and personal determinants are associated with access to and utilization of SRH services in SSA? 2) How do these behaviours and personal determinants contribute to a good quality SRH services provided to women and adolescents in SSA? In this review, behaviours are defined as ways in which healthcare workers act and or conduct themselves towards clients seeking SRH services. Thus, healthcare workers’ behaviours in this study encompass the manner in which a healthcare worker communicates with SRH clients, and any prejudice actions and tendencies unsupportive of SRH and rights of women and adolescents. Personal determinants refers to factors at the individual level, associated with the behaviours of healthcare workers in SRH services which may compromise the quality of the services, and that are potentially changeable by an intervention. Such factors include attitudes, basic knowledge, and skills regarding SRH services.

This systematic review to our knowledge, is the first review focusing especially on healthcare workers’ behaviours and related personal determinants associated with provision of SRH services at large in SSA. It seeks to provide an overview of the behaviours, and related personal determinants of professional healthcare workers in SRH services, which potentially affect adequate provision of SRH services to women and young people. This overview is useful to identify healthcare workers’ behaviours and related personal determinants needing improvement for adequate provision of SRH services. This in turn, will help develop specific interventions that aim to change the unwelcoming healthcare workers behaviours, and ultimately improve access to and utilization of SRH services by those in need, especially young people.

## Methods

To answer the above research questions, a systematic review was undertaken. A protocol, registered with PROSPERO (registration number: CRD42015017509) in advance [21], was developed as a guide to direct the review processes, and can be accessed here: http://www.crd.york.ac.uk/PROSPERO/display_record.asp?ID=CRD42015017509.

### Search strategy

The following electronic databases were searched (from Jan January 1990 until October 2015uary 1990 until October 2015): PubMed, EMBASE (through OVID), Cochrane central (through Cochrane library), CINAHL (through EBSCOhost), and PsychINFO (through EBSCOhost) using a search strategy that was developed based on the key words identified from the study objective, which was adapted according to the technical requirements of each specific database. The full search strategy is provided as Additional file 1.

The search period included studies published since January 1990 until October 2015. This period was chosen because of the MDG 2015 (target 5) pertaining to maternal and child health; which includes amongst others, efforts to reduce maternal mortality, teenage pregnancy, and unmet needs of SRH (family planning) services. Therefore, this review sought to identify studies which were either addressing, or attempting to, or were related to the means and efforts aiming to attain the MDG 5 targets in SRH services. The studies were restricted to: peer-reviewed and academic journals using the specific database filters available and by manual assessment to limit studies to primary studies only (e.g., no editorials, or reviews), studies undertaken in the sub-Saharan African region only, and studies that were published in English language.

### Eligibility criteria

For studies to be included in this review, studies had to pass the eligibility criteria for inclusion: studies with participants as professional healthcare workers, including doctors, nurses, midwives, obstetricians and gyneacologists (*Population*); if the studies were focusing on behavioral and personal determinants of professional healthcare workers, such as education, knowledge and skills of professional healthcare workers in SRH services, studies involving contraceptives, abortion (TOP), pregnancy, childbirth; studies investigating factors associated with access to, utilization of, and quality of SRH services and related services (*Intervention*); if the primary outcomes were quality of healthcare in SRH services, access to SRH healthcare services, utilization of SRH services, and or the secondary outcomes were adolescent pregnancy, maternal health outcomes, and child health outcomes (*Outcome*); if study participants were working in the SRH services, including gyneacology and obstetric care (GOC) services, as well as maternal and obstetric units (MOU); and the studies involving services rendered under general public health facilities, such as primary healthcare clinics (PHC), community health centers (CHC), secondary and tertiary hospitals, as well as academic hospitals (*Setting*). Any types of study designs were eligible for inclusion provided that they reported quantitative data. Only studies with quantitative data focusing on either one or more of the primary and secondary outcomes were eligible. The rationale for the inclusion criteria of only quantitative studies was because this review sought to quantify the relationship between behaviours, determinants, and primary or secondary outcomes in order to inform future intervention development in terms of relevance of specific determinants. Because this review is focusing on determinants rather than comparing interventions or treatment, it does not have a comparator (*No Control*). The full PRISMA checklist is provided as Additional file 2. Two independent reviewers (KJ and RC) performed the selection process of the eligible studies. Studies which do not meet the inclusion criteria according to the independent reviewers were brought forward for discussion and reaching consensus regarding the study’s eligibility for inclusion in this review.

### Review procedure

The search was conducted in a step-wise manner with three phases. The first phase was a broad search that was developed with the guidance of an experienced librarian. The second phase involved searching of specific electronic databases based on the results of the first phase using the provided search terms that were revised and adapted from the first phase. The third and last phase was conducted for additional sources of data. This involved a manual search of reference lists of included reports and articles, and other online resources. Search results were imported into the Endnote database and duplicates were screened for and deleted. Articles went through an extensive screening process in order to identify sources of relevant data.

Initially, titles were scanned for relevance and titles obviously not meeting the inclusion criteria were excluded. Then, the screening of abstracts of those titles that met the inclusion criteria began, and abstracts not meeting the inclusion criteria were excluded. Following the screening of abstracts was the reading of full-text articles of the included abstracts, and articles not meeting all the inclusion criteria were excluded, as well as the articles that did not have full text available. The reasons for exclusion of the full-text articles were either: the article did not report on the behaviours of healthcare workers, or the article reported behaviours based on qualitative findings, or the article reported behaviours of non-professional healthcare workers such as community healthcare workers. Finally, the rigorous and extensive assessment of the included full-text articles as they met the inclusion criteria was conducted. Again, the two independent reviewers (KJ and RC) performed the process described above and met regularly to discuss their assessment until consensus was reached.

**Fig. 1:**
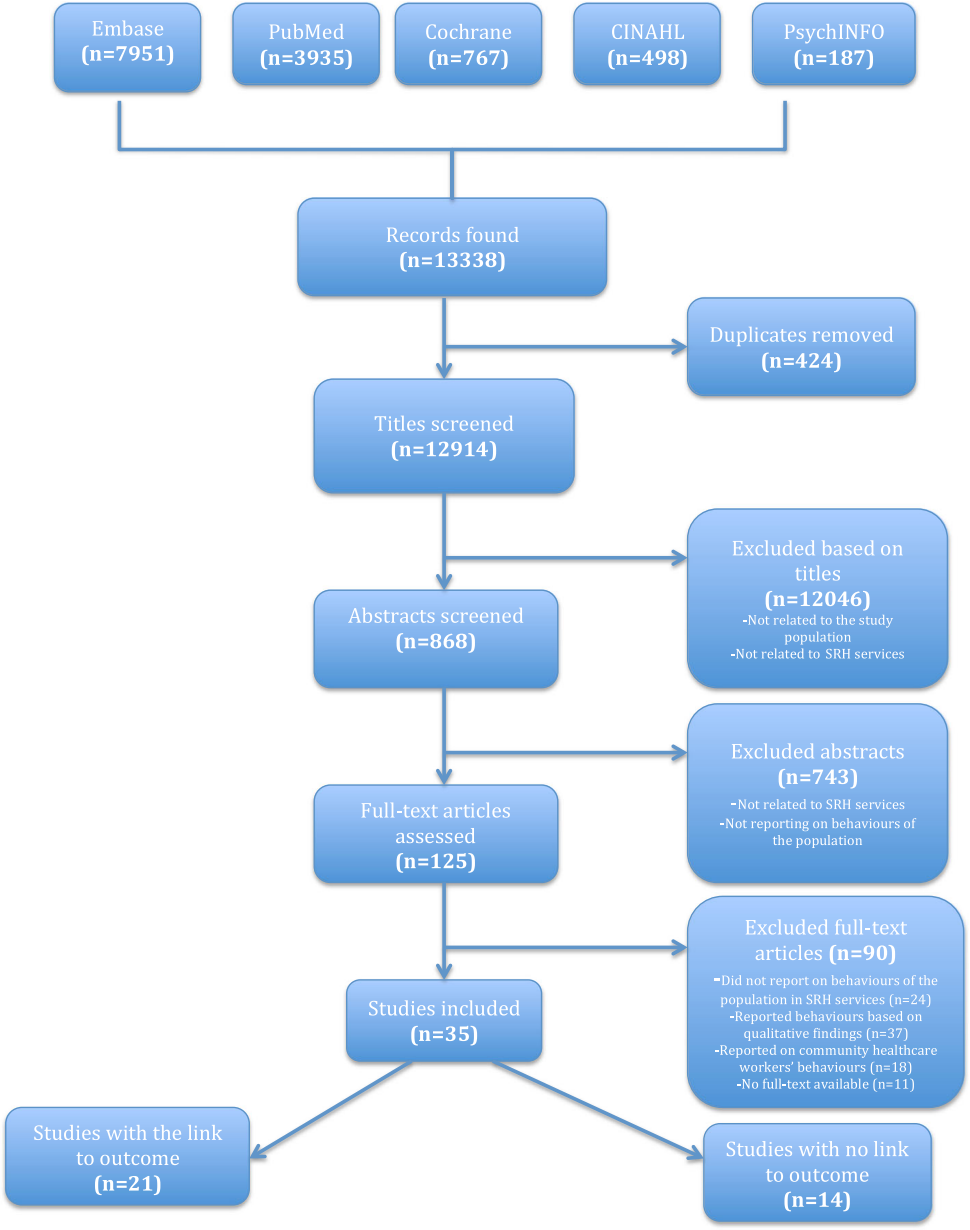
Flow Diagram (PRISMA) showing the different stages of the review process

Experts in the field of maternal and child healthcare, obstetric and gynaecology were also contacted and asked to recommend any new data sources not yet identified through the means described above, including unpublished data. Of the twelve experts contacted via email, only two responded with sources of information consisting of articles not available through the databases searched. The flow diagram below shows the review process from the initial searches of bibliographic databases to the final included studies (see Fig. 1).

### Data abstraction

Publication year, authors, location of study, research design, study participants and outcome measure, as well as data regarding determinants were key factors for data extraction. Data abstraction was carried out by the two independent reviewers (KJ and RC) and they met regularly to discuss their assessment and address any discrepancies until consensus was reached. Where consensus was not reached for a study, the particular study was taken to the next step for further screening and assessment until both reviewers were in consensus. This was also done in order to strengthen the quality of the analysis of the studies included, as well as not immediately excluding studies based on one reviewer’s assessment while studies could potentially be eligible when thoroughly assessed by more than one reviewer. Data from these studies included was extracted and tabulated in Microsoft Excel. Quality assessment of the studies included was assessed using the STROBE checklist for observational studies tool which covers data collection, methods, results, discussion and other information relevant for this review such as the study population. Quantitative data collection methods (e.g., surveys), type of study design (e.g., cross-sectional designs), and description of study participants (e.g., nurses) were among the key criteria in the quality assessment of the studies.

### Data analysis

A narrative analysis approach was used to synthesize the data. For all studies included in this review, data was extracted into a table eliciting information on: (1) first author and year of publication; (2) study sample and population; (3) study design; (4) study outcome; and (5) the determinant(s) with summary measures of the study findings (e.g., attitudes towards SRH services, knowledge of specific SRH components, use of specific SRH resources, and obstetric skills). Furthermore, studies were assessed and data was synthesized for potential to be associated with or impact on the primary or secondary outcomes.

## Results

A total of 13,338 titles were identified through the five electronic databases searched. After removal of duplicates (*n* = 424), 12914 title records were screened and titles not meeting the inclusion criteria were excluded. Screening of the identified titles resulted in 868 abstracts that were reviewed. A total of 125 full articles were then examined for relevance to the review and 35 studies met all the inclusion criteria and therefore are included in this review (see Table 1).

**Table 1:** Main table with all included studies (*n* = 35)

Study/Country	Sample	Design	Outcome	Determinant / summary measures
Tilahun et al. (2012)/Ethiopia [17]	Physician and nonphysician obstetric care workers (*n* = 423)	Cross-sectional study	Attitude toward provision of SRH to unmarried adolescents	Negative attitude toward provision of SRH services to adolescents was significantly associated with healthcare workers’ being married (OR: 2.15; 95% CI 1.44 – 3.06), lower levels of education (OR: 1.45; 95% CI 1.04 -1.99), being a health extension worker (OR: 2.49; 95% CI 1.43 – 4.35), lack of training on RH services (OR: 5.27; 95% CI 1.51 – 5.89), and non-use of family planning (OR: 1.77; 95% CI 1.05 – 2.77).
Okokon et al. (2014)/Nigeria. [22]	Nonphysician obstetric care workers (*n* = 290)	Cross-sectional study	Utilization of the partograph	Knowledge of the partograph had a significant relationship with its utilization in teaching hospital (*Χ* ^2^ = 38.96, p ≤ 0.0001) and general hospital (χ^2^ = 12.05, p ≤ 0.0001), but not in primary healthcare centers (*Χ* ^2^ = 0.08, *p* = 0.778)
Fawole et al. (2008)/ Nigeria [23]	Physician and nonphysician obstetric care workers (*n* = 719)	Cross-sectional study	Knowledge and utilization of the partograph	Utilization of partograph was reported more frequent by responded working in tertiary levels of care compared to those on primary and secondary levels (χ^2^ = 214. 6, p ≤ 0.0001). 37.3% of respondents who were predominantly from the tertiary level of care could correctly mention at least one component of the partograph (χ^2^ = 139.1, p < 0.0001). The partograph is utilized mainly in tertiary health facilities; knowledge about the partograph is poor.
Fawole et al. (2010)/ Nigeria [24]	Physician and nonphysician obstetric care workers (*n* = 275)	Exploratory study	Knowledge of the partograph	Knowledge of partograph components was poor among auxillary nurse/midwives at 0% median score. Knowledge about assessment that could be made by the use of partograph was poor on both settings (private and primary/public care facilities). More responded in the private sector correctly mention the function of the alert line on the partograph than those on the public sector (χ^2^ = 4.43, p < 0.05). Previous training on partograph was associated with knowing at least one component of the partograph (χ^2^ = 49.2, *p* < 0.05). Knowledge about assessment of women during labour was also poor.
Yisma et al. (2013)/Ethiopia [25]	Physician and nonphysician obstetric care workers (*n* = 202)	Cross-sectional study	Utilization of the partograph	Knowledge about partograph was significantly higher among obstetric care workers working in hospitals compared to those working in health centers (OR: 2.0; 95% CI 1.1 – 3.9). However, utilization was significantly more frequent in health canters than in hospitals (χ^2^ = 19.2, *p* < 0.01). Lack of positive attitude toward partograph was associated with poor utilization of the partograph by obstetric care workers (OR: 0.10; 95% CI 0.01 – 0.81). Nonphysician obstetric care workers had lesser likelihood of having a good level knowledge about partograph (OR: 0.10; 95% CI 0.03 – 0.97). Knowledge about partograph was significantly associated with previous training on partograph (OR: 2.80; 95% CI 1.19 – 6.70)
Wakgari et al. (2015)/ Ethiopia [26]	Physician and nonphysician obstetric care workers (*n* = 403)	Cross-sectional study	Utilization of partograph	40.2% utilized partograph during labor. Those who were midwives by profession were about 8 times more likely to have a consistent utilization of the partograph than general practitioners (AOR = 8. 13, 95% CI: 2.67, 24.78). Similarly, getting on job training (AOR = 2. 86, 95% CI: 1.69, 4.86), being knowledgeable on partograph (AOR = 3. 79, 95% CI: 2.05, 7.03) and having favorable attitude towards partograph (AOR = 2. 35, 95% CI: 1.14, 4.87) were positively associated with partograph utilization.
Opiah et al. (2012)/ Nigeria [27]	Nonphysician obstetric care workers (*n* = 165)	Descriptive cross-sectional study	Knowledge and utilization of the partograph	Knowledge of partograph was significantly associated with is utilization ((χ^2^ = 32.29, *p* < 0.05), and between the obstetric care workers’ years of experience and partograph utilization ((χ^2^ = 4.82, *p* < 0.05).
Hussein, et al. (2004)/ Ghana [28]	Physician and nonphysician obstetric care workers (*n* = 416 deliveries)	Retrospective study	Obstetric skills	For all deliveries, 32.6% to 93.0% of criteria were met for “standard care” (defined by Section A criteria), with a mean of 65.5%. No delivery met all of the criteria. Doctors, with a mean score of 76.1%, appear to satisfy more criteria than mid- wives at 67.4%, who in turn satisfy more criteria than assistant midwives, a non-professional grade of staff in private facilities in Ghana.
Nyango et al. (2010)/Nigeria [29]	Nonphysician obstetric care workers (*n* = 54)	Descriptive cross-sectional study	Knowledge and skills among birth attendants	A minority of the obstetric care workers routinely performed basic ANC services. EmOC services provided, including the use of partograph by the respondents were below optimal levels as prescribed by WHO.
Mirkuzie et al. (2014)/Ethiopia [30]	Nonphysician obstetric care workers (*n* = 49)	Health facility-based intervention	Knowledge and skills of basic EmOC	Obstetric care workers’ knowledge on basic EmOC was poor both in 2008 respondents as well as in 2013 respondents.
Ameh et al. (2012)/ Somalia [31]	Physician and nonphysician obstetric care workers (*n* = 222)	Descriptive study	Knowledge and skills of life-saving EmOC, provision of EmOC Availability and quality of EmOC	There was a significant improvement in knowledge (50%) and skills (100%) among the obstetric care workers. Confidence in EmOC provision was improved.
Haile-Mariam et al. (2012)/Ethiopia [32]	Nonphysician obstetric care workers (*n* = 711)	Cross-sectional study	Knowledge of EmOC, resuscitation	Half of the midwives interviewed reported having performed neonatal resuscitation in the past three months compared to only 20% of the nurses. Key predictors of a high knowledge score among providers were recent performance of neonatal resuscitation and geographic region. Whether the provider was a nurse or a midwife, was not associated with a higher knowledge score.
Vivio et al. (2010)/ Zambia [33]	Nonphysician obstetric care workers (*n* = 62)	Observational cross-sectional study	Knowledge of the active management of the third stage labour (AMTSL), provision of AMTSL	Majority of the obstetric care workers knew that AMTSL was associated with the administration of a uterotonic drug. More than half of the participants were aware of the controlled cord tracting (CCT) as a strategy to promote placenta delivery, while a few reported early cord clamping and cutting as a component of the AMTSL protocol, and were observed to use it as a standard practice. A third of the responded were aware of the fundal massage as a component of the AMTSL protocol.
Kimberly et al. (2010)/ Zambia [34]	Nonphysician obstetric care workers (*n* = 21)	Observational study	Knowledge and skills (use) of maternal ultrasound Clinical decision-making	Paired OSCE scores showed a slight overall improvement in the midwives ability to scan at 2 months of 10.0/14, SD 3.9 (71%) and at 6 months of 11.6/14, SD 1.8 (83%). Paired *t*-test showed no significant difference between OSCE 1 and OSCE II with *p* = 0.15
McAuliffe et al. (2013)/ Malawi, Tanzania, Mozambique [35]	Nonphysician obstetric care workers (*n* = 1561)	Cross-sectional study	Intention to leave obstetric services (job satisfaction)	Not receiving any supervision appeared to be most strongly linked to decrements in intentions to leave and job satisfaction. In particular, intentions to leave were substantially increased when no supervision system was in place in Malawi (b = 1.09, SE = 0.31, t = 3.52, *p* < 0.01), Tanzania (b = 0.82, SE = 0.3, t = 2.73, *p* < 0.01), and Mozambique (b = 1.04, SE = 0.39, t = 2.67, *p* < 0.01). There was also clear evidence of a link between the absence of supervision and diminished job satisfaction in all countries. In all three countries we found robust evidence indicating that a formal supervision process predicted high levels of job satisfaction and low intentions to leave.
Maramagi et al. (2004)/ Uganda [36]	Nonphysician obstetric care workers (*n* = 37)	Cross-sectional study	Counseling skills in IMCI programme	Older health providers (50-59 years) were more likely to advise caregivers on medication than younger health providers (χ^2^ = 15.64, *p* = 0.016). Male health providers (χ^2^ = 6.22, *p* = 0.045) and those aged 30-39 years (χ^2^ = 19.244, *p* = 0.004) were more likely to explain the feeding problem to the caregivers. Health providers aged 30 or more years were more likely to give feeding advice than younger health providers (*Χ* ^2^ = 9.62, p = 0.022).
Tita et al. (2006)/ Cameroon [37]	Physician and nonphysician obstetric care workers (*n* = 328)	Cross-sectional study	Awareness of evidence-based obstetric care	A total of 15.5% (50/322) of health workers were aware of all the four interventions while only 3.8% (12/312) reported optimal practice. Evidence-based awareness was strongly associated with practice (PR = 15.4; 96% CI: 4.3–55.0). Factors significantly associated with awareness were: attending continuing education, access to the WHO RHL, employment as an obstetrician/ gynaecologist and working in autonomous military or National Insurance Fund facilities. Controlling for potential confounding, working as an obstetrician was associated with increased awareness (adjusted prevalence odds ratio [aPOR] = 8.3; 95% CI: 1.3–53.8) as was median work experience of 5–15 years (aPOR = 2.0; 95% CI: 1.0–3.8). Internet access was associated with increased practice (aPOR = 3.4; 95% CI: 1.0–11.8).
Ijadunola et al. (2010)/ Nigeria [38]	Physician and nonphysician obstetric care workers (*n* = 152)	Descriptive study	Knowledge of EmOC	Knowledge of EmOC was poor among the respondents, and no difference across age groups. Doctors had a fair knowledge compared to other profession.
Ersdal et al. (2008)/ Zimbabwe [39]	Physician and nonphysician obstetric care workers (*n* = 80)	Cross-sectional study	Knowledge, attitude and practice of symphysiotomy	Seventy-nine of the 80 participants knew about symphysiotomy, and 76 could describe the technique, including 16 of the 17 midwife instructors. One junior doctor was not aware of the intervention. One of the ten obstetricians had occasionally performed a symphysiotomy the other nine did not practice the intervention, but indicated that they would be able to carry it out. All the rural midwives (*n* = 13) regarded symphysiotomy as a lifesaving operation appropriate for remote areas, and 23 of the 39 midwives (59%) thought that the procedure should be taught to midwives.
Ndikom & Onibokun (2007)/ Nigeria [40]	Nonphysician obstetric care workers (*n* = 155)	Cross-sectional study	Knowledge and behaviour towards PMTCT	Nurse/midwives had moderate level of knowledge with mean score of 51.4%. Hypotheses tested revealed that there is a positive relationship between knowledge and behaviour (*r* = 0.583, *p* = 0.00). Knowledge level of nurse/ midwives who had educational exposure was not different from those who did not (t = 1.439, *p* = 0.152). There was a significant difference in the knowledge of nurse/midwives who had experience in managing pregnant women living with HIV/AIDS and those who did not (t = 2.142, *p* = 0.03). Also, there was a significant relationship between behaviour and availability of resources (*r* = 0.318, *p* = 0.000).
Chi et al. (2004)/ Zambia [41]	Physician and nonphysician obstetric care workers (*n* = 225)	Cross-sectional study…	Perceptions toward HIV screening and treatment	Providers reported widespread stigma associated with HIV. Physicians (OR = 1.9), providers with research affiliations (OR = 2.3), and those located in Lusaka (OR = 9.0) were more likely to offer HIV testing. Only 30% routinely prescribed antiretroviral treatment (ART) to reduce MTCT. Practitioners from district facilities, and those employed at research facilities were more likely to prescribe ART routinely (OR = 2.8, 10.1 and 3.4 respectively). Among those never prescribing ART, most cited a lack of availability (83%).
Byamugisha et al. (2007)/ Uganda [42]	Physician and nonphysician obstetric care workers (*n* = 247)	Cross-sectional study	Knowledge, attitude and practice of EC	Most of the participants had heard about EC (79.4%). 1 in 4 (24.1%) of the participants did not know the time limit within which EC is effective. slightly more than half (53.3%) wanted to make the population sensitized about EC issues. About 28% wanted EC available and accessible in convenient places for all. Some HCWs expressed the need for more training, such as seminars in FP methods. Almost half (49%) of the participants who knew about EC had prescribed it in one form or another and 1 in every 10 had prescribed ECPs in the previous 12 months (11.9%).
Traore et al. (2014)/ Mali [43]	Physician and nonphysician obstetric care workers (*n* = 196)	Cross-sectional study	Knowledge and skills of EmOC	Bivariate analysis showed an association between competency score and type of health worker (*p* < 0.05). Knowledge was most deficient for postpartum infection and hypertensive complications. Type of health worker, years of experience, number of days absent, and avail- ability of guidelines for management of obstetric complications within the health center were positively associated with test score (*p* < 0.05). Availability of guidelines was associated with higher competency of physicians, health technicians, and obstetric nurses (*p* < 0.001), and seemed to influence the competency of healthcare workers with fewer than 10 years of experience in particular.
Ehiri et al. (2005)/Nigeria [44]	Nonphysician obstetric care workers (*n* = 252)	Cross-sectional descriptive study	Quality of child health services	Facilities were adequately equipped with immunization supplies. Essential drugs supply was inadequate in all centers; as well as emergency care facilities. 68.3% of the respondents had adequate training in immunization with high knowledge scores on immunization issues. Use of the national case management algorithm was low among the respondents.
Worku et al. (2013)/Ethiopia [45]	Nonphysician obstetric care workers (*n* = 38)	Population-based survey	Availability of MCH services, Obstetric care workers skills, and the quality of MCH services	Availability of essential drugs, equipment and other supplies was unsatisfactory. A small proportion of obstetric care workers had adequate training and experience on important obstetric procedures. Only 24% of the obstetric care workers used partograph frequently and consistently. Majority of the facilities did not function fully for emergency obstetric care (EmOC) as per their level. Important ANC components were incomplete and unsatisfactory.
Mngadi et al. (2008)/ Swaziland [48]	Physician and nonphysician obstetric care workers (*n* = 56)	Exploratory study	Attitude towards adolescents’ SRH service provision	78% respondents reported that they provided contraceptives to the adolescents when they asked for them and when they were available. Some reported that contraceptive use was against their religion and that youth should not indulge in sex. Half of the nurses/midwives had no continued education and lacked supervision on adolescent sexual and reproductive health care. The majority had unresolved moral doubts, negative attitudes, values and ethical dilemmas towards abortion care between the law, which is against abortion, and the reality of the adolescents’ situation. Forty-four wanted to be trained on post-abortion care while eight on how to perform abortions. Twenty-six wanted the government to support adolescent-friendly services and to train healthcare providers in adolescent sexual and reproductive health services.
Oduro-Mensah et al. (2013)/ Ghana [49]	Physician and nonphysician obstetric care workers (*n* = 65)	Cross-sectional descriptive study	Decision making for clinical care	Printed protocols and guidelines were the most commonly selected (96%) aids used in decision-making. However workshop materials (92%), expert advice (90%) and telephone calls for advice (85%) were also frequently selected as aids in daily decision making. The majority of respondents (80%) said they had access to various local (institutionally) modified or developed guidelines and protocols e.g., management of postpartum haemorrhage, management of pre-eclampsia / eclampsia, etc. Health system constraints such as availability of staff, essential medicines, supplies and equipment; management issues (including leadership and interpersonal relations among staff), and barriers to referral were important influences in decision-making.
Leon, Lundgren, & Jennings (2006)/ Rwanda [50]	Nonphysician obstetric care workers (*n* = 40)	Observational study	Guidelines utilization for Provision of contraceptives	Providers implemented less than one third of the guideline set, but they addressed, more frequently than other guidelines, items categorized as essential by expert opinion (*p* <0.01). Rwandan providers emphasized contraindications in 29-min sessions. Within this set, items previously classified as essential were addressed more frequently than guidelines classified as less important in Rwanda (3:2). Use instructions were not addressed frequently than other guideline categories in Rwanda, where contraindications were more prevalent than use instructions. Contraindications ranked above use instructions in both locations in Rwanda.
Warenius et al. (2006)/ Kenya/Zambia [46]	Nonphysician obstetric care workers (*n* = 707)	Cross-sectional study	Attitude towards adolescents’ SRH service provision	Nurse-midwives disapproved of adolescent sexual activity, including masturbation, contraceptive use and abortion, but also had a pragmatic attitude to handling these issues. Those with more education and those who had received continuing education on adolescent sexuality and reproduction showed a tendency towards more youth- friendly attitudes. About two-thirds (69%) of Kenyan and about half (52%) of Zambian respondents disagreed that “16-year-old out-of-school girls should be encouraged to use condoms”. However, both Kenyan (55%) and Zambian (67%) nurse-midwives agreed that “if a schoolgirl was sexually active she should be allowed to use contraceptives”.
Evens et al. (2014)/ Kenya [51]	Nonphysician obstetric care workers (*n* = 20)	Descriptive post-intervention study	Attitude towards post-abortion care (PAC) service and quality of the service provision	Majority of healthcare providers’ were in favour of equal treatment for PAC regardless of age/marital status of clients. All healthcare providers deemed PAC services important. Sexual activity after marriage was supported by 45% of the providers.
Chalmers, McIntyre & Meyer, (1992)/ SA [52]	Physician obstetric care workers (*n* = 203)	Hospital-based survey	Attitude toward caesarean section	Private doctors consider CS safer than a “normal” delivery. Public doctors believe that CS are performed by obstetricians duet of lack of proper (training) management of complication/difficult delivery.
Sidze et al. (2014)/Senegal [47]	Physician and nonphysician obstetric care workers (*n* = 637)	Health facility survey	Access to and use of contraceptives	Obstetric care workers had a minimum age restriction, as well as restrictions according to clients’ marital status, for the provision of contraceptives to young women in Senegal.
Chaibva et al. (2010)/ Zimbabwe [13]	Nonphysician obstetric care workers (*n* = 52)	Descriptive study	Utilization of prenatal services by adolescents	Socio-demographic and cultural factors influenced adolescents’ utilization of prenatal services according to the midwives. Almost all of the midwives (n 1⁄4 49; 94.2%) considered financial constraints to be a factor limiting adolescents’ utilization of prenatal services. 71.2% agreed that health workers’ attitudes could influence adolescents’ decisions. Most midwives (n 1⁄4 45; 86.5%) reported that the quality of prenatal services could influence adolescents’ decisions to utilize prenatal services; 29 (55.8%) felt that adolescents perceived prenatal care to be beneficial because they realised that obstetric problems could be detected and addressed during the prenatal period.
Mane et al. (2014)/Senegal [57]	Physician and nonphysician obstetric care workers (n = 163)	Descriptive study	Provision of emergency contraceptives (EC)	Knowledge gaps about EC among obstetric care workers were found. They also reported reluctance in providing counseling and consequently the service/availability of the service thereof. The workers also carried judgment towards EC users. A larger proportion of the obstetric care workers indicated unwillingness to provide EC to adolescents- only 37% indicated they would provide EC to adolescents who require it.
Lawani et al. (2014)/ Nigeria [58]	Physician obstetric care workers (*n* = 151)	Cross-sectional study	Provision of obstetric analgesia	A total of 74 (49.0%) participants offered obstetric analgesia to parturients in labour that were either non- assisted or assisted with instruments. Among users, only 20 (13.3%) offered obstetric analgesia routinely to parturients, 44 (29.1%) sometimes and 10 (6.6%) on patients’ requests. The comparison between the younger and older age groups was statistically significant with Odds Ratio of 4.37 (1.18-16.18) at 95% CI, *p* = 0.018, while that between urban and rural practitioners was 4.77 (0.99-22.85) at 95% CI, *p* = 0.034.

As shown in the flow diagram of the review process (see Fig. 1.), of the ninety (90) articles excluded, 11 articles were not full-text available, 24 articles did not provide any information on behaviours and attitudes of healthcare workers, 18 articles reported behaviours of non-professional healthcare workers, and the remaining 37 articles reported qualitative findings of behaviours and patients or clients views of healthcare workers’ behaviours and attitudes in SRH services.

### Included studies

A majority of the studies were from Nigeria (*n* = 9) and Ethiopia (*n* = 7), followed by Kenya (*n* = 2), Uganda (*n* = 2), Zambia (*n* = 2), Senegal (*n* = 2), Ghana (*n* = 2), Zimbabwe (*n* = 2), and South Africa (*n* = 2). Malawi, Swaziland, Cameroon, and Rwanda were represented by one study each, and one study represented Mali, Tanzania and Mozambique in one. Of the 35 studies included (see Fig. 1), 21 of them investigated association or influence on the primary outcomes, which affect quality of SRH services and possibly access to and utilization of the services, as described above (see Table 2). Fourteen studies did not investigate this association and only reported on healthcare workers’ behaviours and related factors in SRH services in a descriptive sense.

**Table 2:** Studies associated with the primary or secondary outcomes

Study/Country	Service outcomes			
	Quality of SRH healthcare services	Access (and availability of) to SRH services	Utilization (and provision) of SRH services	Maternal and child health outcomes
Okokon et al. (2014)/Nigeria	✓		✓	
Evens et al. (2014)/ Kenya	✓			
Yisma et al. (2013)/Ethiopia	✓		✓	
Worku et al. (2013)/Ethiopia	✓	✓	✓	
Opiah et al. (2012)/ Nigeria	✓		✓	
Sidze et al. (2014)/Senegal		✓	✓	
Mane et al. (2014)/Senegal		✓	✓	
Ameh et al. (2012)/ Somalia	✓	✓	✓	✓
Ndikom & Onibokun (2007)/ Nigeria		✓	✓	✓
Ehiri et al. (2005)/Nigeria	✓	✓		✓
Tita et al. (2006)/ Cameroon			✓	
Karamagi et al. (2004)/ Uganda	✓	✓	✓	✓
Hussein, et al. (2004)/ Ghana	✓			✓
Lawani et al. (2014)/ Nigeria	✓		✓	✓
Chi et al. (2004)/ Zambia	✓		✓	✓
Wakgari et al. (2015)/ Ethiopia	✓		✓	✓
Fawole et al. (2008)/ Nigeria	✓		✓	✓
Nyango et al. (2010)/Nigeria	✓			✓
Vivio et al. (2010)/ Zambia				✓
Chaibva et al. (2010)/ Zimbabwe			✓	✓
Ersdal et al. (2008)/ Zimbabwe	✓		✓	✓

Of the 35 studies included, 28% (*n* = 10) of them investigated basic obstetric care skills among professional healthcare workers, 23% (*n* = 8) investigated knowledge of SRH service components, 20% (*n* = 7) investigated behaviours of professional healthcare workers with regards to certain SRH services. The remaining 29% (*n* = 10) studies investigated availability of resources and other essential components of SRH services, as factors associated with adequate quality service provision in SRH services. A majority of the studies, 71% (*n* = 25) were cross-sectional studies, some were population and hospital surveys 9% (*n* = 3), exploratory studies 6% (*n* = 2), observational studies 6% (*n* = 2), intervention studies 6% (*n* = 2), and only one was a retrospective study 3% (*n* = 1).

The included studies investigated determinants associated with adequate, quality service provision in sexual and reproductive healthcare (SRH) services and other related factors. The determinants included basic knowledge and skills regarding SRH among professional healthcare workers working in SRH services, as well as their behaviours and attitudes towards providing SRH services. Other factors associated with the behaviours and personal determinants of healthcare workers in SRH, such as the availability of essential SRH resources are also reported. The results are presented under the research questions which this review sought to answer.

### Which healthcare workers’ behaviours and personal determinants are associated with access to and utilization of SRH services in SSA?

In this paper, a description of healthcare workers’ behaviours and personal determinants associated with these behaviours are reported. These behaviours and personal determinants have potential to negatively affect SRH access and utilization of services, as well as quality of care.

### Knowledge and skills of obstetric care in SRH services

Among the studies investigating knowledge, eight of them focused on knowledge of partograph (*n* = 8), a component of basic emergency obstetric care (EmOC), and its association with its utilization in SRH services. These studies were undertaken in Nigeria (*n* = 5), Ethiopia (*n* = 2), and Ghana (*n* = 1) [22–29]. All these studies found that knowledge of partograph had a strong association with its utilization in SRH services. Knowledge of partograph was found to be higher in obstetric care workers working in hospitals than in those working in health centers in Ethiopia [25]. While the knowledge was higher among those working in hospitals, the utilization was more frequent in the health centers than in hospitals [25]. The knowledge of partograph components was found to be poor among the auxillary nurses and midwives in Nigeria [24]. More years of experience of a healthcare worker with the healthcare system in Nigeria was associated with better partograph utilization in terms of frequency and skills of partograph use [27].

A majority of the studies (*n* = 10) investigated knowledge of other components of basic EmOC and found: basic EmOC knowledge among the obstetric care workers was poor, EmOC knowledge and skills and confidence in providing the services was improved by training, higher knowledge scores of some life-saving techniques, such as neonatal resuscitation were associated with having performed the technique recently and with geographical location [30–39]. However, type of healthcare worker such as being a nurse or a midwife did not have any associations with higher knowledge scores among healthcare workers in Ethiopia [32].

Knowledge of active management of the third stage of labour (AMTSL) was fairly adequate among the healthcare workers in Zambia, although a few of the workers still practiced cord clamping and cutting, a procedure no longer recommended as part of AMTSL [23]. Only a third of healthcare workers were aware of the fundal massage as a component of AMTSL [33]. The knowledge and use of maternal ultrasound among midwives in Zambia was improved after training on maternal ultrasound [34]. Knowledge of basic EmOC among the different professions in SRH services in Nigeria was better among doctors compared to other professions, but no difference was found between age groups [38]. Knowledge of symphysiotomy, a procedure performed to widen the pelvis in order to ease childbirth when there is a mechanical problem, was moderate among all participants in the Zimbabwean study and all could describe how to perform it [39]. In particular, rural midwives in Zimbabwe regarded the technique as a lifesaving one appropriate for remote areas, and more than half of them thought that it was necessary to be taught [39].

With regards to PMTCT, knowledge among nurses and midwives was moderate Nigeria, with knowledge and behaviour being positively correlated [40, 41]. Knowledge of PMTCT among nurses and midwives who manages pregnant women living with HIV and AIDS in Nigeria was significantly better from those who did not manage pregnant women living with HIV and AIDS [40]. Knowledge of emergency contraceptive, on the one hand, was fair among the different cadre of healthcare workers in Uganda [42]. On the other hand, 1 in 4 healthcare workers did not know the time limit within which EC was effective. Knowledge of postpartum infections and management of hypertensive complications was fairly poor among the healthcare workers in Mali [43]. However, high knowledge scores on immunization issues were found among healthcare workers in Nigeria [44].

With regards to continuous education, a model often used in the healthcare setting to continuously improve knowledge and skills of healthcare workers, nearly half of the participants in a study in Swaziland had no continuous education training and felt that they needed guidance or supervision on adolescent sexual and reproductive health care, although some of them wanted to be trained on post-abortion care while some wanted to be trained on how to perform abortions [45]. A small proportion of the healthcare workers wanted the government to train healthcare workers in adolescent sexual and reproductive health services [45]. In Nigeria however, a large proportion of healthcare workers (68%) had adequate training in immunization, according to Ehiri et al. [44].

### Attitudes toward women and adolescents seeking SRH services

Negative attitudes towards provision of SRH services to adolescents in Ethiopia was significantly associated with healthcare workers’ being married, lower levels of education, being a health extension worker, lack of training on RH services, and non-use of family planning by healthcare workers themselves [17]. A majority of healthcare workers in both Kenya (82%) and Zambia (86%) reported that they would advise adolescents to abstain from sex when they seek contraceptives [46]. With regards to provision of contraceptives to young women, healthcare workers had a minimum age restriction, as well as restrictions according to clients’ marital status in Senegal [47]. However, healthcare workers with more education and those who had received continuing education on adolescent sexuality and reproduction showed a tendency towards more youth- friendly attitudes and were in support of contraceptive use among adolescents [46]. Furthermore, these two countries concurred with the notion of adolescent boys being taught the dangers of masturbation [46]. On the positive attitudes of healthcare workers, a majority of the obstetric care workers in Zimbabwe agreed that adequate knowledge about prenatal services, accessible and acceptable services, affordable services, needs-focused prenatal services, prompt services and perceived benefits would influence women and adolescents’ decisions to utilize prenatal services [13].

### Healthcare workers’ cultural and religious beliefs regarding SRH services

Religion was found to be a factor among healthcare workers in Swaziland as some healthcare workers reported that contraceptive use was against their religion and that youth should not indulge in sex [48]. Furthermore, masturbation is regarded as a sinful act and those who perform it are believed to experience mental disorders or fertility problems by a majority of healthcare workers in both Kenya (*n* = 264) and Zambia (*n* = 331) [46]. In general, masturbation is considered a taboo in the southern region of SSA. Nurses and midwives disagreed that abortion should be provided to adolescent girls with unwanted pregnancies, on both cultural and religious grounds in Kenya (80%) and Zambia (94%) [46]. Nearly a third (26%) of the nurses and midwives in both countries reported that they would feel annoyed if an adolescent girl would report with abortion-induced related symptoms [46].

### Availability of SRH services and resources

In Ethiopia, availability of essential drugs, equipment and other supplies was unsatisfactory, and discrepancies were found between providers and clients with regards to services provided and services received [45]. A majority of the facilities in Ethiopia did not function fully for EmOC as per their level, as well as important ANC components were incomplete and unsatisfactory [45]. Ndikom & Onibokun (2007) [40] found a significant relationship between behaviours and availability of resources. While facilities were adequately equipped with immunization supplies in Nigeria, essential drugs supply was inadequate in all centers; as well as emergency care facilities [40]. Various healthcare system constraints, such as human resources, availability of essential medical supplies including drugs, and challenges with the referral system were reported as key factors influencing decision-making for clinical care in Ghana [49].

Printed protocols and guidelines were the most commonly selected aids used in decision-making for clinical care and the availability of guidelines was associated with higher competency of physicians, health technicians, and obstetric nurses [49]. However, study participants also reported healthcare systems’ facilitators in decision-making for clinical care in Ghana. The facilitators included availability and consultation of workshop materials, expert advice and telephone calls to other colleagues, and access to various locally (institutionally) modified or developed guidelines and protocols e.g., guidelines on the management of postpartum [49].

Contrary to Oduro-Mensah, et al., [49], Implementation of clinical guidelines was less than optimal, with certain sections of the guidelines better implemented than others by healthcare workers in Rwanda [50]. Furthermore, higher competency in EmOC skills was linked to the availability of guidelines which further enhanced competency of other healthcare workers with fewer than 10 years of experience in Mali [43].

### Other factors associated with provision of quality SRH services

The major factors found to influence behaviour towards PMTCT services were mainly fear of getting infected, irregular supply of resources like gloves, goggles, and sharps boxes, and an irregular supply of water [40]. A majority of healthcare workers in Kenya were in favour of equal treatment for post abortion care (PAC) regardless of the age or marital status of clients [51]. All healthcare workers in Kenya deemed PAC services important. Sexual intercourse after marriage was supported by less than half of the healthcare workers in the Kenyan study [51]. Private doctors in South Africa considered caesarean section (CS) safer than a “normal” delivery, a convenience and financially enticing to the doctor [52]. Public doctors believed that CS is performed by obstetricians due to a lack of proper (training) management of complications or difficult delivery in South Africa [52].

## Discussion

This systematic review includes studies on the behaviors and personal determinants of healthcare workers pertaining to sexual and reproductive health from sub-Saharan Africa. Negative behaviours and attitudes of healthcare workers, as well as personal determinants, such as poor knowledge and skills of SRH services, and related factors, like unavailability of essential drugs and equipment are associated with provision of inadequate SRH services. Negative behaviours, including negative attitudes of healthcare workers regarding some SRH services, particularly for adolescents’ SRH services were commonly reported in the studies included in this review [17, 46, 51, 52]. Healthcare workers’ negative behaviours and attitudes are unlikely to encourage women in general to access and utilize SRH services, but more specifically young women.

With regards to adolescents specifically, it appears that nurses and midwives disapprove of adolescents’ sexual activities and show reluctance in providing SRH services to adolescents who engage in sexual activities [46]. Healthcare workers’ negative behaviours and attitudes towards adolescents seeking SRH services, such as contraceptives are well documented in SSA, and have a negative effect on adolescents’ access to and utilization of the services [11, 13, 16, 17, 46, 48, 51–53]. Consequently, many adolescents continue to fall pregnant and for some, the pregnancy is unwanted [1, 47, 48, 54]. Furthermore, negative behaviours and attitudes of healthcare workers considerably affect women and adolescents’ decision to access and utilize antenatal care services during pregnancy, including decisions to deliver the baby at a healthcare facility and post-natal care thereafter [13, 53]. This finding is a cause for concern, as contraceptive use by adolescents is needed to help reduce teenage pregnancy, and antenatal care is important to monitor pregnancy and helps to detect and prevent potential complications during pregnancy.

Evidence shows that healthcare workers’ negative behaviours discourage women and adolescents from accessing and utilizing SRH services [11–20]. For example, healthcare workers’ negative attitudes towards provision of contraceptives to adolescents hinder adolescents’ access to and utilization of SRH services [17, 18]. It is therefore clear that interventions which aim to address and change the negative behaviours and attitudes of healthcare workers in SRH services are likely to improve healthcare utilization, especially by adolescents.

Personal determinants, which most consistently appeared to be associated with adequate quality provision of SRH services at large, were knowledge and skills of SRH service components, and continuous education on SRH service components. Knowledge and skills of basic, yet necessary and life-saving components of SRH services is unsatisfactory, according to the studies included in this review. Continuous education, a method of keeping abreast with current and updated knowledge also appears to be a method least utilized by healthcare workers according to the studies included. This is concerning, given the poor knowledge and skills of SRH services among healthcare workers reported by the studies included in this review.

Knowledge of specific SRH components, such as the knowledge of partograph and the components of active management of the third stage of labour (AMTSL) are below optimum levels, and are not being fully implemented by some healthcare workers as per the WHO recommended guidelines of EmOC and AMTSL. These SRH components include the poor utilization of partograph during labour, the controlled cord clamping and cutting, and the fundal massage, which are all very useful and can save lives if fully and correctly implemented. This finding echoes findings from a study by Ueno et al., [55] which investigated and evaluated the implementation of EmOC components in public hospitals where only two hospitals fully implemented the nine components of EmOC. Signal functions requiring skill like manual removal of placenta, removal of retained products, or vacuum extraction, were also not performed at all in the healthcare centre, and only three out of eight facilities provided anticonvulsants even though eclampsia is one of the leading causes of complications during labour and of maternal deaths [55].

These components are crucial components of adequate and quality SRH services, and are essential to mother and child healthcare outcomes. Therefore, optimum knowledge and skills of both EmOC and AMTSL are necessary to improve maternal and child health, and reduce maternal and child mortality and morbidity. Furthermore, evidence synthesized in this review show that healthcare workers have limited knowledge of emergency contraceptives (EC) and how it works. This is also a cause for concern as knowledge of EC and its provision is particularly important to prevent unwanted or unplanned pregnancies. This finding is similar to previous research, where knowledge of EC was relatively poor among healthcare workers [42, 54, 56, 57]. Adolescents are the most frequent group of people who tend to use and or need EC to prevent unwanted pregnancies after having had unsafe sex [54]. However, if healthcare workers do not know how EC work and when it is best to provide it, it is unlikely that they would encourage adolescents to seek this service or provide it should the need arise [56–58]. This further puts limitations for adolescents to access and utilize services for their SRH needs.

Healthcare systems related factors, such as a lack of resources including essential drugs and equipment, also have a relation to inadequate quality provision of SRH services. Inadequate availability of essential drugs and other equipment not only impede access to and utilization of services, but also the provision of adequate quality service in SRH services. Factors such as a shortage of staff, drugs and equipment, and limited skills have been identified in the literature as possible reasons why quality of SRH services is not optimal, for example EmOC requiring skills are the least performed by healthcare workers in SRH services [39, 50]. Thus, to improve quality service provision, and access to and utilization of services in SRH, sufficient availability of resources is crucial.

It is worth noting that SSA countries are different from each other, but also share a few similarities, such as poor infrastructure, limited healthcare resources, including limited human resources in healthcare settings. With regards to culture and religion, some countries in SSA are more sensitive than others. For example, Swaziland is more sensitive to religion than culture and thus, behaviours of many healthcare workers in this country is informed or based on their religious beliefs. While in SA and Ethiopia for example, culture and traditional practices are more value and inform the behaviours of most healthcare workers [17, 18, 48]. Both the differences and similarities provide useful information when designing and developing interventions to improve the negative behaviours of healthcare workers in SSA countries. Furthermore, these differences and similarities helps in identifying countries where maximum efforts should be placed, and which specific behaviours should be prioritized when designing the interventions for improvement.

### Limitations

Many of the studies included came from northern regions compared to the southern regions of SSA, although it is well known that inadequate SRH services are predominantly vast in the southern region. Methodologically, the majority of the studies included in this review were descriptive cross-sectional studies with a strong heterogeneity. This makes it hard to pool results from different studies. Moreover, it is possible that some interesting findings are in the grey literature, which this review did not access. The other limitation is the lack of information on adolescent specific obstetric care. However, this is not surprising, as obstetric care in general has been tailored around the general population of women and not adolescents.

The results of this review are not necessarily generalizable to each and every country in the SSA region due to the limited number of studies supporting this review’s findings. Hence, caution is warranted to draw general conclusions. However, the limited number of studies in SRH services in SSA emphasizes the need for more research in this field in order to draw conclusive and generalizable findings. Furthermore, findings of this review can be used to some extent to highlight specific areas needing intervention, but additional needs assessment maybe needed. It is clear that more research still needs to be done to uncover healthcare workers’ behaviours and associated factors which have a negative effect on access to and utilization of SRH, and quality of care in this field.

### Implication for practice

Healthcare workers behaviours, personal values, and morale significantly hinder the provision of adequate quality SRH services to young people, especially to adolescent girls. Adolescents’ SRH services need to be youth-friendly, and served by welcoming healthcare workers who have a positive attitude towards adolescents seeking SRH services. This kind of environment will further equip adolescents seeking SRH services with the knowledge and information they require to make informed choices regarding their SRH.

It has been shown that interactive forms of continuous education sessions on sexual and reproductive healthcare that enhance participant activity and provide the opportunity to practice skills can effect change in professional practice and, improve health care outcomes [59, 60]. The findings in this review of doctors having better knowledge of specific SRH components compared to nurses and midwives, highlights the importance of further education and training among nurses and midwives in providing SRH services.

Basic EmOC signal functions, as described by the WHO, cannot be fully performed if a health facility is inadequately equipped with necessary resources to carry all the signal functions. The availability, continuous and frequent utilization of SRH resources, such as the partograph are very important and cannot be over emphasized as they play a crucial role in maternal and child health outcomes. Therefore, increasing essential supplies and equipment in health facilities is very important to strengthening the healthcare system and improving health outcomes.

### Implication for policy

Continuous SRH education and training is necessary to adequately provide quality services, and therefore needs to be incorporated and enforced into the healthcare systems policies for professional development. The importance of comprehensive knowledge and skills to adequately care for women, newborn, children, and adolescents should be prioritized as it is critical in SRH services. Reducing maternal morbidity and mortality rates, including deaths of newborn, and children under five years of age, are a priority in the SDG 2016-2030. To ascertain that this specific target is achieved in SSA, serious efforts and investments towards adequate quality healthcare services are needed.

Religion, a socio-demographic factor associated with many behaviours, is commonly acknowledged in the SSA region and requires attention to assist healthcare workers who find themselves in conflict of their religion when rendering some SRH services. Healthcare workers in SRH services may benefit from policy guidelines and instructions pertaining to religious beliefs and clarifying the role of healthcare providers in this regard. Healthcare workers should be able to provide SRH services to women and adolescents when they require them, irrespective of their own personal views and religious beliefs.

### Implications for research

There is a need for an in-depth understanding of the negative behaviours and attitudes of healthcare workers in SRH services, and their associated factors in order to develop interventions that will address them and consequently stimulate access to and utilization of SRH services by women and young people in SSA. Interventions to promote positive behaviours and attitudes of healthcare workers in SRH services are needed. Furthermore, future studies should be intervention-based focusing on changing the known and reported negative behaviours and attitudes of healthcare workers in SRH services into positive ones. Such intervention studies may include changing the negative behaviours and attitudes of healthcare workers with regards to young women’s use of contraceptives in some parts of SSA, taking into account the contextual differences in SSA regions. An SRH specific evaluation tool to regularly monitor and evaluate knowledge and skills of healthcare workers in SRH services would be useful to ensure continuous education and training, and improved SRH knowledge and skills.

## Conclusions

Knowledge of SRH services, including basic EmOC is insufficient among healthcare workers in SSA. Healthcare workers also seem to experience a conflict between their attitudes towards the sexual behaviors and their role as providers of support and information about SRH issues to adolescents in particular. The more knowledgeable the healthcare worker is the better is his/her behaviour. Positive behaviours and attitudes are highly likely to influence women and adolescents’ utilization of SRH services for their needs. Healthcare workers in SRH services have to be clinically trained and skilled on comprehensive sex education and other related SRH issues for women and adolescents specifically. Access to and utilization of adequate, quality SRH services will most likely reduce teenage pregnancy and the illegal and unsafe abortions in the SSA region. A continuous education program for healthcare workers in SRH services can be feasible and potentially successful, given the interest expressed by healthcare workers.

## Additional files

Additional file 1: Search Strategy. (PDF 58 kb)
Additional file 2: PRISMA checklist. (PDF 115 kb)

## Abbreviations

AIDS: Advanced immune deficiency syndrome
AMTSL: Active management of the third stage of labour
ANC: Antenatal care
CHC: Community healthcare centers
CS: Caesarean section
EC: Emergency contraceptives
EmOC: Emergency obstetric care
GOC: Gyneacology and obstetric care
HIV: Human immunodeficiency virus
LMIC: Low- and middle-income countries
MDG: Millennium development goals
MOU: Maternal and obstetric units
PAC: Post abortion care
PHC: Primary healthcare center
PMTCT: Prevention of mother-to-child transmission
SDG: Sustainable development goals
SRH: Sexual and reproductive health
SSA: Sub-Saharan Africa
STI: Sexually transmitted infection
