# Supplementary material for: Healthcare workers’ behaviors and personal determinants associated with providing adequate sexual and reproductive healthcare services in sub-Saharan Africa: a systematic review
Source: BMC Pregnancy Childbirth. 2017 Mar 13;17:86. doi: 10.1186/s12884-017-1268-x (PMC5348841; doi:10.1186/s12884-017-1268-x)
Supplement: Additional file 1: — Search Strategy. (PDF 58 kb) [file 12884_2017_1268_MOESM1_ESM.pdf]

Search Strategy

The following electronic databases were searched (from Jan January 1990 until October 2015uary 1990 until October 2015): PubMed, EMBASE (through OVID), Cochrane central (through Cochrane library), CINAHL (through EBSCOhost), and PsychINFO (through EBSCOhost) using a search strategy that was developed based on the key words identified from the study objective, which was adapted according to the technical requirements of each specific database. The full search strategy is provided as Additional file 1.

The search period included studies published since January 1990 until October 2015. This period was chosen because of the MDG 2015 (target 5) pertaining to maternal and child health; which includes amongst others, efforts to reduce maternal mortality, teenage pregnancy, and unmet needs of SRH (family planning) services. Therefore, this study sought to identify studies which were either addressing, or attempting to, or were related to the means and efforts aiming to attain the MDG 5 targets in SRH services. The studies were restricted to: peer-reviewed and academic journals using the specific database filters available and by manual assessment to limit studies to primary studies only (e.g., no editorials, or reviews), studies undertaken in the sub-Saharan African region only, and studies that were published in English language.
